# Co-inoculations of bacteria and mycorrhizal fungi often drive additive plant growth responses

**DOI:** 10.1093/ismeco/ycae104

**Published:** 2024-08-07

**Authors:** Louis Berrios, Andressa M Venturini, Tillson Bertie Ansell, Esther Tok, William Johnson, Claire E Willing, Kabir G Peay

**Affiliations:** Department of Biology, Stanford University, 327 Campus Drive, Stanford, CA 94305, United States; Department of Biology, Stanford University, 327 Campus Drive, Stanford, CA 94305, United States; Department of Biology, Stanford University, 327 Campus Drive, Stanford, CA 94305, United States; Division of CryoEM and Bioimaging, SSRL, SLAC National Accelerator Laboratory, Menlo Park, CA 94025, United States; Department of Biology, Stanford University, 327 Campus Drive, Stanford, CA 94305, United States; Oceans Department, Hopkins Marine Station of Stanford University, 120 Ocean View Blvd., Pacific Grove, CA 93950, United States; School of Environmental and Forest Sciences, University of Washington, Seattle, WA 98195, United States; Department of Biology, Stanford University, 327 Campus Drive, Stanford, CA 94305, United States; Department of Earth System Science, Stanford University, Stanford, CA 94305, United States; Woods Institute for the Environment, Stanford University, Stanford, CA 94305, United States

**Keywords:** bacteria-mycorrhizal fungi interactions, tripartite interactions, meta-analysis, bioinoculants, microbial ecology

## Abstract

Controlled greenhouse studies have shown the numerous ways that soil microbes can impact plant growth and development. However, natural soil communities are highly complex, and plants interact with many bacterial and fungal taxa simultaneously. Due to logistical challenges associated with manipulating more complex microbiome communities, how microbial communities impact emergent patterns of plant growth therefore remains poorly understood. For instance, do the interactions between bacteria and fungi generally yield additive (i.e. sum of their parts) or nonadditive, higher order plant growth responses? Without this information, our ability to accurately predict plant responses to microbial inoculants is weakened. To address these issues, we conducted a meta-analysis to determine the type (additive or higher-order, nonadditive interactions), frequency, direction (positive or negative), and strength that bacteria and mycorrhizal fungi (arbuscular and ectomycorrhizal) have on six phenotypic plant growth responses. Our results demonstrate that co-inoculations of bacteria and mycorrhizal fungi tend to have positive additive effects on many commonly reported plant responses. However, ectomycorrhizal plant shoot height responds positively and nonadditively to co-inoculations of bacteria and ectomycorrhizal fungi, and the strength of additive effects also differs between mycorrhizae type. These findings suggest that inferences from greenhouse studies likely scale to more complex field settings and that inoculating plants with diverse, beneficial microbes is a sound strategy to support plant growth.

## Introduction

Bacteria and mycorrhizal fungi colonize the roots of nearly all land plants [1, 2], and their impact on plant growth and health can range from deleterious to beneficial [3, 4]. Although several studies have used them as inoculants to assess plant responses such as changes to plant biomass, mycorrhizal colonization, and shoot height [5–7], most studies often use single inoculations (i.e. either bacteria or mycorrhizal fungi). Considering that other tripartite investigations have revealed, for example, that some non-mycorrhizal fungi can suppress phytopathogenic microbes and thereby enhance plant growth, it is likely that the interactions between soil bacteria and mycorrhizal fungi also interact in ways that shape plant growth responses (see [8] for a comprehensive review). Greenhouse studies have indeed shown that bacterial inoculations can increase mycorrhizal colonization, which tends to increase plant growth and vigor [9, 10], whereas others have shown that some soil bacteria deter the growth of mycorrhizal fungi [11]. Evidence from field studies has likewise illustrated that soil bacteria and mycorrhizal fungi often have strong, predictable interactions [12–14] and can benefit plant growth by warding off pathogens, mobilizing nutrients, and producing phytohormones [15, 16]. It is therefore clear that bacteria and mycorrhizal fungi interact and consequently shape various plant growth responses, but their individual and combined effects on plant growth responses remain less clear [17]. As such, a comprehensive framework for assessing these tripartite interactions would benefit the field of plant–microbe interactions, particularly when trying to bridge the gap between greenhouse and field studies.

To accurately predict how belowground bacterial–fungal interactions affect plant growth and health, it is first critical to determine the type of effects that these organisms generate. We know that biotic interactions can yield both additive and nonadditive effects (i.e. higher order interactions—HOIs). Additive effects are defined as those that equal the sum of their parts. The addition, for example, of either “Microbe A” *or* “Microbe B” to “Plant 1” may increase or decrease plant biomass by 2-fold (relative to an uninoculated plant). An additive response would, therefore, result if the addition of both “Microbe A” *and* “Microbe B” increases or decreases plant biomass by the sum of responses to individual inoculations (e.g. 4-fold). In contrast, a nonadditive effect or HOI would result if the addition of “Microbe A” and “Microbe B” caused plant biomass to change by a factor either significantly more or less than four (i.e. by a factor that is unequal to the sum of responses to individual inoculations). Identifying and parsing these two divergent effects also have large-scale implications. Several reports, for instance, have shown that the inclusion of additive and higher order effects in statistical models clarifies our understanding of tropical tree growth [18], ecosystem responses to global change [19], and stressor effects in freshwater ecosystems [20]. Likewise, HOIs have been shown to impact species removal, species diversity, and community responses to multiple stressors in natural ecosystems [21, 22], indicating that nonadditivity plays a crucial role in both the selection of organisms and organismal stability in natural environments [23–26]. Yet, microscale ecological processes both drive and respond to macroecological processes, and the high degree of spatial overlap between mycorrhizal fungi and rhizosphere bacteria suggests that there should be strong interactions between them that could play a major role in determining observed plant growth responses. A quantitative evaluation of these interactions and their degree of additivity has, however, not been carried out yet.

In addition to identifying the general type of interactions that often occur between bacteria, mycorrhizal fungi, and plants, the strength, direction, and frequency of interactions between microbial symbionts and plant hosts also have many important implications for scalability. First, these features are necessary to scale findings from simple greenhouse experiments to complex field environments [27]. The synergistic or antagonistic interactions among symbiotic root microbes are seldom investigated, despite evidence suggesting that microbial interactions have strong effects on soil microbial communities [28]. Second, these features determine whether agriculture and conservation efforts can benefit from applying multiple microbes (or removing specific microbes) to optimize plant responses [29]. Lastly, they function as a metric to assess and reinforce lab-to-field translation. That is, if there are specific microbes with strong, positive effects on plant performance in the lab [14, 30], can these microbes then be assumed to have similar effects in field environments that harbor different bacteria and fungi?

Although several studies have qualitatively reviewed bacterial–fungal interactions [6, 31, 32], quantitative studies that address these interactions have not been reported. Since meta-analyses are an effective approach for uncovering quantitative trends across many individual studies with varying methodologies [33–36], we conducted a meta-analysis to address the type, frequency, direction, and strength of plant responses (i.e. total plant biomass, shoot biomass, root biomass, shoot height, root length, and mycorrhizal colonization) to either single inoculations (bacteria or mycorrhizal fungi) or co-inoculations (bacteria and mycorrhizal fungi). In addition, we investigated these plant responses within two dominant guilds of mycorrhizal fungi—arbuscular mycorrhizal (AM) and ectomycorrhizal (EcM) fungi—to determine shared and divergent features of bacterial–mycorrhizal fungi interactions. In total, our analyses included 82 studies that involve AM fungi and 22 studies that involve EcM fungi, which collectively include more than 60 plant genera, more than 40 bacterial genera, and more than 20 genera of mycorrhizal fungi. Together, our results suggest that bacteria and mycorrhizal fungi primarily generate positive, additive effects on plant growth responses and that, as a result, scaling plant performance predictions from simple to complex communities is feasible.

## Materials and methods

### Study selection

**Figure 1:**
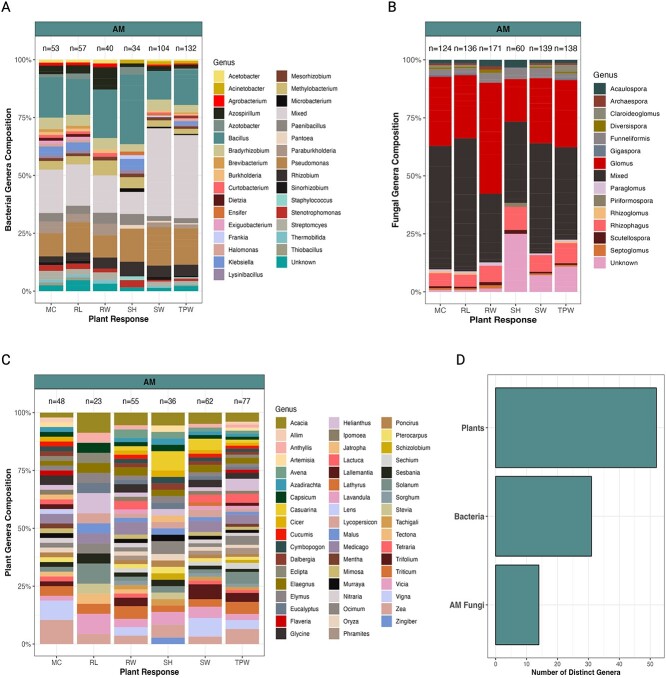
Distribution of organisms used in AM studies. The composition of bacterial (A), fungal (B), and plant (C) genera of studies used in our meta-analysis is shown. The *x*-axis displays the six plants responses that were analyzed in our study. The abbreviations are as follows: MC = mycorrhizal fungi colonization percentage, RL = plant root length, RW = plant root weight, SH = plant shoot height, SW = plant shoot weight, and TPW = total plant weight. The total number of inoculants used in each analysis is denoted above each stacked bar. See Supplementary Table S1 for additional information about the selected studies. (D) Number of unique genera across studies.

**Figure 2:**
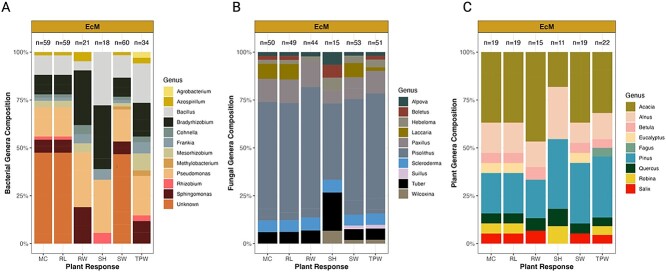
Distribution of organisms used in EcM studies. The composition of bacterial (A), fungal (B), and plant (C) genera of studies used in our meta-analysis is shown. The *x*-axis displays the six plants responses that were analyzed in our study. The abbreviations are as follows: MC = mycorrhizal fungi colonization percentage, RL = plant root length, RW = plant root weight, SH = plant shoot height, SW = plant shoot weight, and TPW = total plant weight. The total number of inoculants used in each analysis is denoted above each stacked bar. See Supplementary Table S1 for additional information about the selected studies.

To understand how bacteria and mycorrhizal fungi impact plant responses, we collected plant response data from a total of 104 studies (see Supplementary Table S1 and Figs 1 and 2 for a full list of summary statistics) and compared the plant response effect sizes across inoculation types (i.e. bacteria alone, fungi alone, bacteria plus fungi). In February of 2023, articles with the following keywords were downloaded from Web of Science: “bacteria AND ectomycorrhizal fungi AND plant growth AND inoculation” and “bacteria AND arbuscular mycorrhizal fungi AND plant growth AND inoculation.” This search generated a total of 930 studies (230 studies that included EcM fungi and 700 studies that included AM fungi). To facilitate statistical tests, we filtered our dataset to include only experimental studies that (1) had at least four conditions (i.e. plant alone, plant with mycorrhizal fungi, plant with bacteria, and plant with mycorrhizal fungi and bacteria), (2) had at least three replicates per condition, and report either (3) plant biomass, shoot height, root length, or (4) percent mycorrhizal colonization. In many studies, we found either (3) and (4), but not both in a single study. However, we report the number of studies used for each analysis in Supplementary Table S1 and the number of samples per analysis in Figs 1 and 2. Note that these totals represent studies that satisfy both (1) and (2) and either (3) or (4). A few studies observed mycorrhizal colonization in non-mycorrhizal controls with only the addition of single bacterial inoculations, suggesting that these studies may have had unintended mycorrhizal fungal spores in their bacterial single inoculation condition(s) or perhaps some enhancement of ambient mycorrhizal contamination**.** Since these studies were so few and had insignificant effects on mycorrhizal colonization status compared to treatments intended to have mycorrhizal fungi inoculants, we left them in our analyses as ecologically conservative controls. However, we removed studies that had a similar percentage of mycorrhizal colonization in control conditions (i.e. plants not intentionally inoculated with mycorrhizal fungi) compared to mycorrhizal inoculations, since high levels of contamination make it difficult to accurately gauge treatment effects. Studies that reported mycorrhizal status (i.e. EcM vs. AM) inaccurately (e.g. reporting non-EcM fungi as EcM fungi) were likewise removed. After filtering our dataset, we retained a total of 22 studies with EcM fungi and 82 studies with AM fungi (Supplementary Table S1).

### Data analysis

We organized and analyzed our dataset in R [37]. The means from all plant responses were extracted either directly from tables or figures in published articles (Supplementary Table S1). Plant weight data were converted to grams, and plant or root length measurements were converted to centimeters. The plant, bacterial, and fungal organisms used in each study were recorded and are reported in Supplementary Table S1. The organisms used in studies were grouped by genus and are represented in Figs 1 and 2. Taxonomy is reported according to the nomenclature conventions used at the time of publication, and we did not attempt to resolve changes in nomenclature (e.g. *Funneliformis* vs. *Glomus*) in part to ease the tractability of articles for readers. Soil chemistry data were not reported frequently enough to be included in our analyses.

To facilitate comparisons across conditions, we calculated the effect sizes for each plant response (i.e. total plant weight, root weight, root length, shoot height, shoot length, and colonization percentage) as previously described by Hoeksema *et al.* [34]. Briefly, the log ratio of inoculated plants (experimental condition) to the uninoculated plants was calculated as ln(X_i_/X_n_), where X_i_ is the mean plant response in an inoculated treatment, and X_n_ is the mean plant response in an uninoculated control. Therefore, the effect size is positive for beneficial interactions that improved plant growth responses and negative for detrimental interactions that decreased plant growth responses relative to controls. We used this log response ratio instead of other effect size metrics because it yields a standardized and unitless measure of plant responses across studies, which makes them the ideal choice for meta-analyses [38]. Since each of our plant responses included one control and three experimental conditions, this approach yielded three effect sizes (i.e. bacteria alone, fungi alone, bacteria plus fungi) that were relativized to the control conditions. However, in cases where effect sizes would equal infinity (due to control conditions yielding a value of zero, e.g. percent mycorrhizal colonization), we replaced control values with a value of 1 to calculate responses.

Statistical analyses were performed in R [37]. To test for differences between groups, we performed pairwise *t*-tests using the stat_compare_means function in the *ggpubr* package. Because the purpose of this study was to compare the effects of bacterial and fungal single and co-inoculations on plant growth responses, we do not include direct statistical tests between control and experimental conditions. However, microbial inoculants tended to have a net positive effect on measure plant growth responses (i.e. positive effect sizes). Linear regression models were also generated using the *lm* function to estimate the impact that input predictor variables (e.g. inoculation type: bacteria alone, fungi alone, and bacteria plus fungi) had on plant responses (e.g. plant biomass and mycorrhizal colonization). If significant interaction terms (i.e. *P*<.05 for the bacteria X fungi term) were observed between bacteria and fungi, we classified these interactions as higher order interactions (HOIs) or nonadditive relationships [22, 25]. In contrast, if no significant interactions were observed between bacteria and fungi, then their relationships were classified as additive. We are also aware that others [23, 24, 26] have adopted slightly different definitions of nonadditivity, but for the purposes of our analyses, this was the most operationally useful approach. In addition, we generated standardized model residuals versus leverage plots (Supplementary Figs S1 and S2) to test for patterns of publication bias. Influential data points that fell outside of Cook’s distance (0.5) were then removed to reduce possible biases in the results. All graphs were generated using either base R or *ggplot* [37, 39].

## Results

### Patterns in the taxonomic selection of bacteria, mycorrhizal fungi, and plants

To understand the taxonomic distribution of organisms used in this field, we grouped bacterial, fungal, and plant taxa by genus and calculated their frequency across studies (Figs 1 and 2). In total, 31 bacterial genera were used in AM studies, and 11 bacterial genera were used in EcM studies (Figs 1A and 2A). A significant proportion of bacteria used in both AM and EcM studies were in the genera *Bacillus* and *Pseudomonas*. However, AM studies often used known, mixed bacterial consortia, whereas many EcM studies used unknown bacterial inoculants (i.e. not taxonomically classified; Supplementary Table S1). The fungal organisms used across studies were slightly less diverse compared to their bacterial counterparts. A total of 14 AM fungal genera were used in AM studies—*Glomus* being the most dominant, aside from a large number of mixed AM fungi inoculants (Fig. 1B). In comparison, EcM studies were comprised of 11 EcM genera, and they often used the genus *Pisolithus* (Fig. 2B). Regarding the plant genera that were used as hosts, AM studies included a total of 52 plant genera, and *Acacia* and *Zea* were the most common plant genera (Fig. 1C). In contrast, EcM studies included a total of 10 genera—with *Acacia* and *Pinus* comprising ~50% of all the plant taxa (Fig. 2C). Together, these data demonstrate that the literature on the interactions between bacteria, mycorrhizal fungi, and plants has used a relatively diverse group of bacteria and plants but a more restricted group of mycorrhizal fungi. As such, efforts to expand beyond common taxa (e.g. *Bacillus*, *Glomus*, and *Acacia*), detail the exact bioinoculants used in experiments (i.e. avoid using unknown inoculants), and report the identities of mixed inoculants (i.e. more than one bacterial or fungal strain) will help advance our understanding of how these organisms interact.

**Figure 3:**
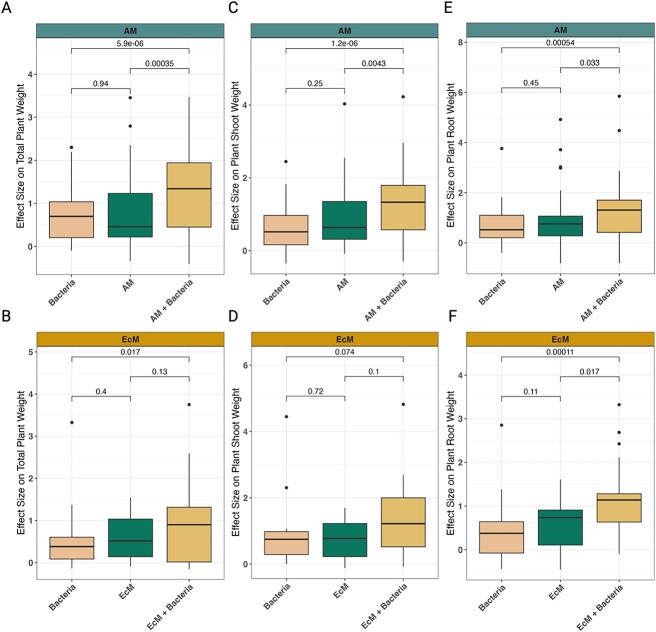
Biomass plant response effect sizes of single and co-inoculations of bacteria and mycorrhizal fungi. Changes in effect sizes (*y*-axis) for total plant weight (A and B), plant shoot weight (C and D), and plant root weight (E and F) are shown for both AM fungi and EcM fungi across different inoculation types (*x*-axis). The *P*-values for each comparison are provided, where *P*<.05 is considered a significant difference. Study information can be found in Figs 1 and 2 and Supplementary Table S1. The linear regression model outputs are listed in Table 1, Supplementary Tables S2, and S3.

**Figure 4:**
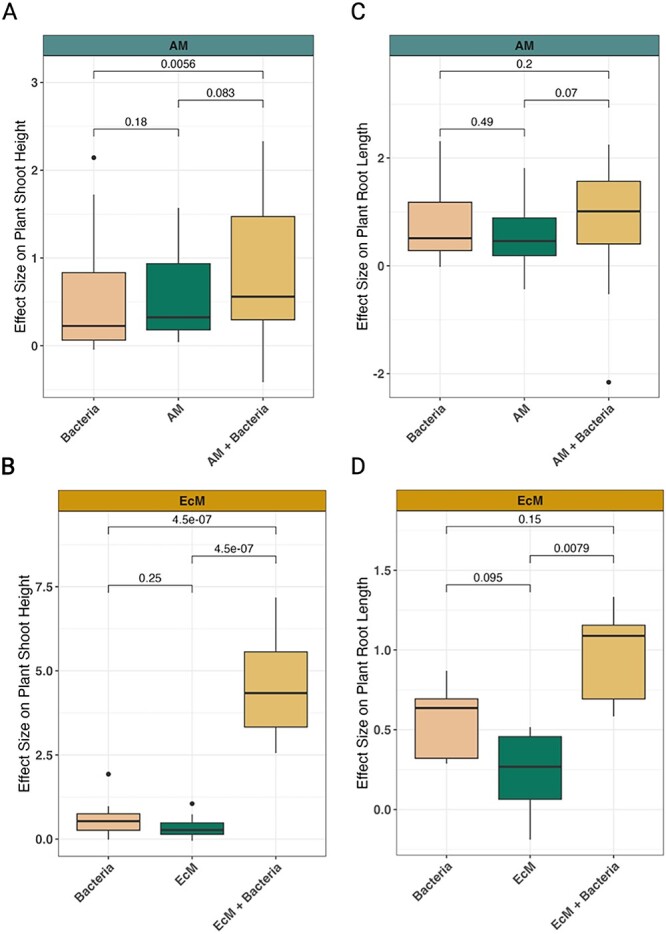
Plant shoot height and root length effect size comparisons of single and co-inoculations of bacteria and mycorrhizal fungi. Changes in effect sizes (*y*-axis) for plant shoot height (A and B) and plant root length (C and D) are shown for both AM fungi and EcM fungi across different inoculation types (*x*-axis). The *P*-values for each comparison are provided, where *P*<.05 is considered a significant difference. Study information can be found in Figs 1 and 2 and Supplementary Table S1. The linear regression model outputs are listed in Table 1, Supplementary Tables S2, and S3.

### The addition of bacteria usually boosts the positive effects that mycorrhizal fungi have on several plant growth response measurements

**Figure 5:**
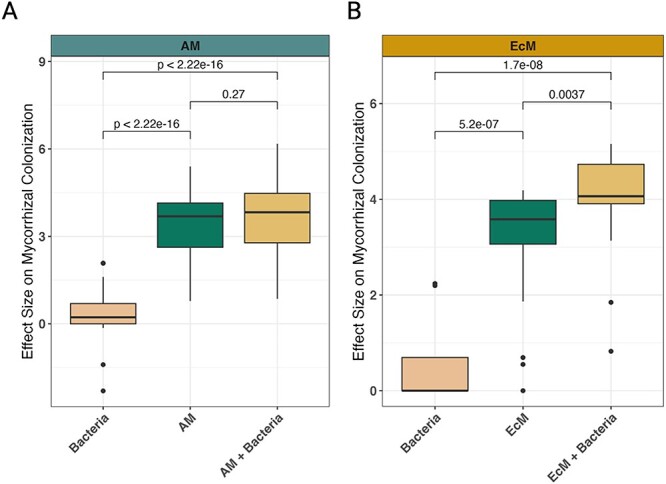
Mycorrhizal fungi plant root colonization percentage effect size comparisons of single and co-inoculations of bacteria and mycorrhizal fungi. Changes in effect sizes (*y*-axis) for the colonization of AM fungi (A) and EcM fungi (B) across different inoculation types (*x*-axis) are shown. The *P*-values for each comparison are provided, where *P*<.05 is considered a significant difference. Study information can be found in Figs 1 and 2 and Supplementary Table S1. The linear regression model outputs are listed in Table 1, Supplementary Tables S2, and S3.

When we analyzed how microbial inoculations affected plant responses, we found that both single and co-inoculations of bacteria and mycorrhizal fungi often had a positive and significant effect. The effects, however, varied depending on the type of plant response and fungal guild (AM or EcM). For example, though many responses to single inoculations were similar (Figs 3 and 4), co-inoculations of AM fungi and bacteria caused total plant biomass to increase significantly compared to single inoculations (Fig. 3A). Studies using EcM plants, however, showed that co-inoculations of bacteria and EcM fungi only significantly increased total plant weight more than that of bacterial single inoculations—not single EcM fungi inoculations (Fig. 3B). When we analyzed the effects on plant shoot and root weight, we found that co-inoculations of bacteria and mycorrhizal fungi (AM and EcM) increased shoot and root weight beyond that of single inoculations (Fig. 3C–F). For plant shoot height and plant root length, co-inoculations of bacteria and EcM fungi were the only inoculation type to have significant effects, and these were considerably more responsive in EcM plants compared to AM plants (Fig. 4). Similarly, we observed that co-inoculations caused mycorrhizal root colonization of EcM fungi—but not AM fungi—to significantly increase relative to single fungal inoculations (Fig. 5). Together, these results indicate that the aspects of plant growth that respond most to bacterial inoculation vary between AM and EcM fungi, but in general plant growth is maximized when bacteria are used in conjunction with mycorrhizal fungi.

### Both additive and nonadditive bacterial–mycorrhizal fungi interactions drive plant growth responses

Next, we constructed linear regression models to determine whether interactions between bacteria and mycorrhizal fungi generate additive or nonadditive effects (i.e. HOIs) on plant responses. If we observed a significant interaction term between predictor variables (i.e. presence or absence of bacterial and fungal inoculants) on effect size response variables (e.g. total plant weight), then these were classified as HOIs. Otherwise, the relationships between bacteria and fungi were classified as additive. Of the six plant responses we analyzed, we observed additive effects for five responses and HOIs for one response (i.e. plant shoot height in EcM plants). Though additivity dominated most of the responses that we measured, the strength of the interaction terms for bacteria and AM fungi compared to bacteria and EcM fungi differed substantially (Table 1; Supplementary Tables S2 and S3). Moreover, all AM fungi models were weaker in their predictive power compared to EcM fungi models. For example, the presence of bacteria and fungi only explained 28% of the effect size variance for shoot height in AM plants, whereas models predicting EcM shoot height explained 86% of input data. In addition, single inoculations of AM fungi were significant predictors of shoot height, but single inoculations of EcM fungi were not significant predictors of shoot height (Table 1). In sum, these analyses demonstrate that nonadditive effects (i.e. HOIs) are rare among commonly reported plant phenotype responses to bacterial and mycorrhizal fungi co-inoculations, whereas additive or “sum of their parts” responses predominate.

**Table 1:** Linear regression model outputs of the effect that bacterial and mycorrhizal fungal inoculants have on plant responses.

**Fungal Guild**	**Response**	**Predictor**	**Estimate**	**Std. Error**	**t value**	***P* value**
**AM**	Shoot height	Bacteria	5.30E-01	1.07E-01	4.947	1.83E-06
		Fungi	5.54E-01	1.31E-01	4.234	3.79e-05^*^^*^^*^
		Bacteria × fungi	-2.15E-01	1.73E-01	−1.243	.216
	Total plant weight	Bacteria	7.06E-01	1.00E-01	7.028	1.40e-11^*^^*^^*^
		Fungi	7.65E-01	1.10E-01	6.966	2.06e-11^*^^*^^*^
		Bacteria × fungi	−1.86E-01	1.50E-01	−1.245	.214
	Shoot weight	Bacteria	6.06E-01	1.09E-01	5.54	7.77e-08^*^^*^^*^
		Fungi	8.49E-01	1.22E-01	6.958	3.10e-11^*^^*^^*^
		Bacteria × fungi	−1.67E-01	1.65E-01	−1.012	.312
	Root weight	Bacteria	6.61E-01	1.48E-01	4.473	1.23e-05^*^^*^^*^
		Fungi	8.85E-01	1.61E-01	5.5043	1.00e-07^*^^*^^*^
		Bacteria × fungi	−3.47E-01	2.21E-01	−1.571	.118
	Root length	Bacteria	7.71E-01	1.96E-01	3.945	.000169^*^^*^^*^
		Fungi	5.56E-01	2.21E-01	2.514	.013923^*^^*^^*^
		Bacteria × fungi	−4.13E-01	2.99E-01	−1.381	.170962
	Mycorrhizal colonization %	Bacteria	4.36E-01	1.50E-01	2.9	.00403^*^^*^^*^
		Fungi	3.34E+00	1.53E-01	21.819	<2e-16^*^^*^^*^
		Bacteria × fungi	−1.28E-01	2.20E-01	−0.584	.55951
**EcM**	Shoot height	Bacteria	5.61E-01	2.57E-01	2.187	.0333^*^^*^^*^
		Fungi	3.38E-01	2.57E-01	1.318	.1933
		Bacteria × fungi	3.60E+00	3.82E-01	9.431	9.15e-13^*^^*^^*^
	Total plant weight	Bacteria	4.57E-01	1.38E-01	3.305	0.00121^*^^*^^*^
		Fungi	5.38E-01	1.41E-01	3.806	0.00021^*^^*^^*^
		Bacteria × fungi	−7.20E-02	1.99E-01	−0.362	.718
	Shoot weight	Bacteria	6.42E-01	2.05E-01	3.139	.002246^*^^*^^*^
		Fungi	8.42E-01	2.14E-01	3.937	.000155^*^^*^^*^
		Bacteria × fungi	−1.67E-01	2.97E-01	−0.563	.574923
	Root weight	Bacteria	4.12E-01	1.62E-01	2.537	.012762^*^^*^^*^
		Fungi	5.77E-01	1.68E-01	3.44	.000856^*^^*^^*^
		Bacteria × fungi	1.24E-01	2.32E-01	0.532	.596186
	Root Length	Bacteria	5.62E-01	1.47E-01	3.836	.00132^*^^*^^*^
		Fungi	2.23E-01	1.47E-01	1.525	.14572
		Bacteria × fungi	1.86E-01	2.12E-01	0.877	.39285
	Mycorrhizal colonization %	Bacteria	4.14E-01	2.59E-01	1.598	.114
		Fungi	3.15E+00	2.59E-01	12.175	<2e-16^*^^*^^*^
		Bacteria × fungi	4.14E-01	3.68E-01	1.125	.264

Statistically significant predictors and/or interaction terms are denoted by ^*^ for *P*<.05 and ^*^^*^^*^ for *P*<.01. Model output data were generated using the summary() function on each constructed model in R. DF, degrees of freedom. See Supplementary Tables S2 and S3 for additional information about the model outputs.

## Discussion

Individually, plant-associated bacteria and mycorrhizal fungi play pivotal roles in helping plants establish and survive across the globe [40–43]. However, a body of evidence has emerged over the past decade, showing that strong patterns of co-occurrence exist between specific bacterial taxa and mycorrhizal fungi—even across large environmental gradients [14, 44], and these microbial interactions can shape nutrient economies for each other and their plant hosts [45]. Yet, we still lack a clear understanding of how their combined interactions shape plant growth responses. This is a particularly important gap in the literature to address because most studies explicitly investigate either bacteria–plant or fungi–plant interactions, which can lead to incomplete predictions of how plants develop in complex environments. Determining whether these microbial relationships generate additive or nonadditive effects (i.e. HOIs), for instance, can help clarify not only the rates that plants grow but also the type of investment (e.g. more shoot or more root biomass) that plants are likely to undergo in nature—all of which can lead to enhanced predictions of plant growth, biomass allocation, abiotic and biotic stress responses, and the impact of climate-related stressors. Therefore, studies that ask how bacteria and mycorrhizal fungi both singularly and concertedly shape plant responses promote balance between mechanistic insight and ecological realism. To this end, we collected plant response data from studies that used single and co-microbial inoculations and conducted a meta-analysis to identify the general interaction types between bacteria and mycorrhizal fungi and determine their impact on common plant responses. Our results demonstrate that interactions between bacteria and mycorrhizal fungi often drive positive and additive plant responses, but the interactions between bacteria and EcM fungi generate nonadditive effects on the height of EcM plants. The implications of our work, therefore, recalibrate our understanding of how underground biotic interactions shape aboveground processes for two of the most prevalent mycorrhizal plant groups on Earth.

### Magnified in the literature: Easy to culture equals commonly used

The organisms that have been used to assess bacteria–mycorrhizal fungi–plant interactions to date have been phylogenetically diverse (Figs 1 and 2), but a heavy reliance on a few taxa has likely limited our understanding of these complex interactions. For instance, both AM and EcM studies used mainly bacterial inoculants from the genus *Bacillus* and *Pseudomonas* (Figs 1A and 2A), and close to 25% of all the bacterial inoculants used were either mixed cultures (many of which belonged to Rhizobia groups; see Supplementary Table S1) or unknown inoculants (Fig. 2A). The fungal inoculants were likewise dominated by a few genera (i.e. *Glomus* for AM studies and *Pisolithus* for EcM studies) or known, mixed cultures and unknown inoculants (Fig. 1B). The fact that these bacterial and fungal taxa are often straightforward to isolate in pure culture and represent common soil and plant root associates [46, 47] explains their repeated use in studies. But their impact on plant growth responses in complex, adaptive systems (e.g. forests and agricultural lands) may be minimal compared to other taxa given that they represent only a small fraction of the microbial diversity that persists in soils. Nevertheless, both rare and abundant taxa can have significant impacts on soil and plant health, and it will be important moving forward to understand how the addition of focal species impacts soil community composition [17]. Moreover, communicating the microbial taxa used (and their respective input concentration), which were too often not reported in studies (Figs 1 and 2), severely hinders our understanding of how plants and microbes interact because it impedes scientific reproducibility [40, 48, 49]. In contrast to the microbial inoculants used across studies, the plant genera used were considerably more diverse, particularly in AM studies (Figs 1C and 2C). A total of 52 plant genera were used in AM studies, and a total of 10 plant genera were used in EcM studies—which highlights that our findings here are likely generalizable features of interactions among bacteria, mycorrhizal fungi, and plants (at least in terms of how plants respond to these microbes). However, studies on bacteria–EcM fungi interactions still only summed to about one-fourth the amount of bacteria–AM fungi studies, suggesting that more EcM studies should be conducted. This point becomes even more critical when considering that EcM fungi are significantly more diverse than AM fungi at both local and global scales [50, 51].

### Why do we observe mycorrhizae-specific differences?

Our results demonstrate that co-inoculations with bacteria and mycorrhizal fungi tend to enhance many plant biomass parameters beyond that of single inoculations (Figs 3 and 4). Yet, differences between mycorrhizal type (i.e. AM vs. EcM) were observed. For instance, the effect of bacteria on mycorrhizal colonization appears to be neutral for AM plants (though a statistically insignificant increase was observed) and positive for EcM plants (Fig. 5). How interactions occur between bacterial cells and fungal spores could explain these different colonization responses. For instance, bacterial metabolites such as auxofuran have been shown to enhance EcM fungal spore germination [52], whereas the volatile 2-methylisoborneol was correlated with AM fungal spore germination [53]. However, plant-derived metabolites can also enhance fungal spore germination [54, 55], and how bacteria produce, consume, or modulate these metabolites in the context of fungal spore germination and colonization remains unclear.

One of the most parsimonious explanations for the observed differences in mycorrhizal root colonization—along with root length, shoot height, plant biomass, and shoot weight (Figs 3 and 4)—are likely linked to ecophysiological differences between AM and EcM fungi. That is, EcM fungi encapsulate plant roots, forming a hyphal sheath but do not penetrate plant cell walls, whereas AM fungi penetrate plant cell walls and interact directly with plant cell membranes [56]. The fact that AM fungi (but not EcM fungi) penetrate cell walls suggests that they may select for different—both qualitatively and quantitatively—bacterial communities than EcM fungi. This could result in changes to the rate and quality of bacterial-mediated nutrient acquisition for both AM and EcM plants—a key feature of many bacterial-mycorrhizal fungi interactions [5, 12, 57]. In line with this idea is the notion that host-microbe immune recognition processes may differ between AM and EcM fungi, their bacterial communities, and their host plant [17, 58, 59], which could prime plant hosts for symbioses in divergent ways and subsequently change plant growth outcomes. The differences in host recognition and symbiosis maintenance may be further increased given that EcM fungi occupy more physical space and access more soil organic matter than AM fungi, which may create a larger habitat with greater selection for bacterial specialization [60]. Efforts to therefore gauge the molecular crosstalk between bacteria, mycorrhizal fungi, and plants across time would help clarify the different responses that we observed.

Another explanation of mycorrhizae-specific differences that deserves attention is the relatively small number of EcM plant species used across experiments compared to AM studies. AM studies included about five times more plant species than EcM studies (Figs 1 and 2). A similar study to ours [34] nonetheless found that mycorrhizal responsiveness is relatively conserved to the plant family level, which supports the notion that the small number of EcM plants used in our analyzed studies likely imparted little bias to the overall effects that we observed. Agricultural and economic incentives alongside shorter plant growing periods likely explain this experimental bias between the number of AM and EcM plant species used to date, but it cannot be ignored that species-specific interaction strengths may exist. For example, the common use of *Acacia* species—know N-fixers—in EcM studies may change the types of bacterial–fungal–plant relationships that occur underground, considering N-fixing plant hosts tend to be less responsive to mycorrhizal inoculations [34]. As such, a subset of efforts should focus on expanding the EcM species used in tripartite experiments (e.g. N-fixers and non-N-fixing plants) and incorporating plants that form both AM and EcM symbioses. Together these efforts will help uncover the general and specific mechanisms that explain interactions among bacteria, mycorrhizal fungi, and plants.

### Why do bacteria and mycorrhizal fungi often generate additive plant growth responses?

Why exactly additive effects prevail over nonadditive effects remains an outstanding question. The answer likely depends on the type of bacterial–fungal interaction (positive, neutral, negative), the extent to which bacteria and mycorrhizal fungi provide the same vs. different benefits, and the plant response curve (i.e. linear vs. nonlinear) to these benefits. It could be that bacteria and mycorrhizal fungi simply operate under independent yet complementary mechanisms (i.e. positive additivity) or that the benefits of one microbe are cancelled out by the costs of another microbe (i.e. neutral additivity) [61]. In interactions between AM fungi and root herbivores, for example, the increased nutrient uptake that AM fungi provide grassland plants was cancelled out by the negative effect of root herbivores—an observation that the authors attribute to functional dissimilarity between soil groups [62]. In our study, however, most of the additivity was positive, suggesting that bacteria and mycorrhizal fungi support plant growth through complementary mechanisms, such as access to distinct forms of the same nutrient (e.g. organic vs. mineral N). In contrast to additive responses, nonadditive or nonlinear plant responses may be the result of competitive, antagonistic processes, where microbes normally help the host but limit each other’s ability to provide benefits to the host when together, such as through antibiotic production or competition for host space. Positive interactions between bacteria and mycorrhizal fungi also arise through changes to plant nutrients or hormones that inherently have nonadditive responses to one another. For example, a meta-analysis found synergistic effects in >50% of studies that applied simultaneous N and P addition [63], which they suggest could result from nutrient co-limitation. Similarly, bacteria and mycorrhizal fungi have been shown to modulate the plant hormones brassinosteroid and gibberellin, which play key roles in shoot height development and exhibit a molecular crosstalk dialogue that may promote nonadditive plant responses [64–66]. Since our study showed that bacteria and EcM fungi generated positive, nonadditive effects on plant shoot height, the product of these microbial interactions may therefore alter the expression of genes or hormones that support shoot height and development [61, 67]. Likewise, the products of bacteria–EcM fungi interactions may also cause multi-level changes to mechanisms involved in xylem-specific conductivity, leaf size, leaf area, wood density and modulus of elasticity—which all affect plant energy investments to shoot development [68]. However, it remains unclear how active or abundant these soil microbes are throughout plant development and how their interactions impact plant gene expression or hormonal regulation in the context of plant health. The mechanisms that undergird both additive and nonadditive processes will become clearer from efforts that assess both plant and microbial responses in tandem. Much research is therefore still required to fully understand how these emergent properties manifest and why different mycorrhizal plants (i.e. AM vs. EcM) and different plant growth traits have varied responses to bacterial and mycorrhizal fungi co-inoculations.

### From basic ecology to commercialization: Could the answer lie in the “right” combination?

Efforts to commercialize bioinoculants have remained constant over the past few decades [69, 70]. Although these efforts have gained moderate success [71], many bioinoculants fail to work in complex environments such as agricultural fields and forest soils [72]. Our analyses show that (as opposed to single microbial inoculations) co-inoculations of bacteria and mycorrhizal fungi may improve the efficacy of existing bioinoculants. Given that the experiments we analyzed included only a single plant host, it is possible that the observed effects of co-inoculations may not hold up in complex plant communities (i.e. outside of monoculture agriculture or forestry), but our results align well with the fact that microbial diversity tends to have positive effects on terrestrial ecosystems and that bacterial–fungal interactions can determine soil health and benefit plant growth [14, 73, 74]. Efforts that investigate how bacteria and mycorrhizal fungi interact within mixed mycorrhizal communities (i.e. harboring both AM and EcM fungi hosts at varying densities) and how the strength of mycorrhizal fungi plant host dependence may alter bacterial–fungal interactions would help test the notion that above- and belowground complexity may alter simple tripartite interactions. In line with this, investigations in diverse forest types (e.g. temperate versus tropical or old growth versus young forests) and differing agricultural lands (e.g. soil chemistry, hydrological, and cropland differences) will be critical moving forward. Similarly, effectively implementing bacteria and mycorrhizal fungi co-inoculations for land management purposes will require detailed analyses that identify the mechanisms of these tripartite relationships in the context of priority effects and their evolutionary history [75].

Although our analyses begin to shed light on ways to improve current formulations of bioinoculants [76, 77], each experiment that we analyzed was conducted in ambient or ideal conditions with little or no fertilizer added, which does not address how climate change will impact the effectiveness of applied microbial inoculants nor how differing land management factors may impact tripartite symbioses. The diversity and abundance of mycorrhizal fungi, for example, are predicted to decline in some regions of the globe, with evidence suggesting that soil phosphorus limitation may influence responses of mycorrhizal fungi to climate change. This coupled with the fact that fertilizer amendments (which vary in composition and usage) are known to affect plant–microbe interactions [78–82] calls into question how these factors then affect bacterial–fungal interactions and their relationships with plant communities in field settings. A key step toward enhancing the effectiveness of bioinoculants will be to therefore identify which pairings of microbes, or which communities, can be effectively applied across various environmental contexts and global change factors. Likewise, developing our understanding in the context of current agricultural (e.g. till vs. no-till or heavy pesticide vs. organic farming) and forestry practices (e.g. burn practices) will also be critical for the success of bioinoculants, and applying large-scale field experiments in these contexts will be imperative to both our fundamental and applied knowledge in this field [83].

## Conclusion

Soils are the most microbially diverse habitat on Earth [30], but until now it has been difficult to generalize the interaction type, strength, and direction of bacterial–fungal interactions and how they relate to plant growth responses. Our analyses demonstrate that bacteria and mycorrhizal fungi often generate additive plant responses, though microbial HOIs do occur. This information will not only strengthen predictions of large-scale processes from small-scale experiments, but it can also be used to help guide land management and conservation practices. Likewise, this information provides a framework for understanding how these interactions and the species that generate them might be impacted in the face of climate change.

## Supplementary Material

Supplementary_Data_7-11-24_ycae104

Table_S1_ycae104

## Data Availability

All data generated or analyzed during this study are included in this published article and its supplementary information files.
