# Supplementary material for: Co-inoculations of bacteria and mycorrhizal fungi often drive additive plant growth responses
Source: ISME Commun. 2024 Aug 7;4(1):ycae104. doi: 10.1093/ismeco/ycae104 (PMC11346365; doi:10.1093/ismeco/ycae104)
Supplement: Supplementary_Data_7-11-24_ycae104 [file supplementary_data_7-11-24_ycae104.pdf]

Supplementary Data

Co-inoculations of bacteria and mycorrhizal fungi often drive additive plant growth responses

Louis Berrios✉[1], Andressa M. Venturini[1], T. Bertie Ansell[1,2], Esther Tok[1], William Johnson[3], Claire E. Willing[4], Kabir G. Peay[1,5,6]

[1] Department of Biology, Stanford University, Stanford CA, 94305, USA
[2] Division of CryoEM and Bioimaging, SSRL, SLAC National Accelerator Laboratory, Menlo Park CA, USA
[3] Oceans Department, Hopkins Marine Station of Stanford University, 120 Ocean View Blvd., Pacific Grove, CA, 93950, USA
[4] School of Environmental and Forest Sciences, University of Washington, Seattle WA, 98195, USA
[5] Department of Earth System Science, Stanford University, Stanford CA, 94305, USA
[6] Woods Institute for the Environment, Stanford University, Stanford CA, 94305, USA

✉ Lead Contact: Louis Berrios; Email: berriosl@stanford.edu

The supplementary data here includes Table S2, Table S3, Figure S1, and Figure S2. Table S2 and Table S3 include additional linear regression model output data, which pairs with data presented in Table 1 of the main text. Likewise, Figure S1 and Figure S2 show the standardized residual by leverage plots to accompany regression model data presented in the main text.

Table S1. Raw data of studies analyzed. This table includes raw plant response data, inoculant conditions, and study titles and DOIs. These data can be found as a separate Excel file, due to its large size.

Table S2. Linear regression full model output statistics. Statistically significant predictors and/or interaction terms are denoted by * for p < 0.05 and *** for p < 0.01.

Fungal Guild	Plant Response	F-Statistic$_{DF}$	Residual Standard Error	Adj. R Squared	p-value
**AM**	Total Plant Weight	$54.643_{3,301}$	0.6468	0.3461	< 2.2e-16
	Shoot Weight	$47.1_{3,246}$	0.6426	0.3571	< 2.2e-16
	Root Weight	$22.94_{3,224}$	0.8265	0.2248	5.479e-13
	Shoot Height	$23.05_{3,167}$	0.5373	0.2802	1.543e-12
	Root Length	$8.437_{3,81}$	0.6767	0.2099	6.048e-05
	Mycorrhizal Colonization %	$303.9_{3,276}$	0.9156	0.7651	< 2.2e-16

**EcM**	Total Plant Weight	$15.26_{3,141}$	0.5979	0.229	1.185e-08
	Shoot Weight	$14.21_{3,97}$	0.7445	0.2839	9.469e-08
	Root Weight	$16.4_{3,98}$	0.5855	0.3138	1.037e-08
	Shoot Height	$107.5_{3,51}$	0.7009	0.8554	< 2.2e-16
	Root Length	$16.35_{3,17}$	0.2419	0.6972	2.945e-05
	Mycorrhizal Colonization %	$115.5_{3,85}$	0.8678	0.7961	< 2.2e-16

Table S3. Linear regression model outputs. In each model, the response variable was the effect size, and the predictor variables were bacterial, fungal, or bacterial plus fungal inoculants. Model test statistics are not enclosed by parentheses, while CI 95% statistics are enclosed by parentheses. N = Number of observations. Significance codes are as follows: *** p < 0.001; ** p < 0.01; * p <0.05. Plant growth parameters are also as follows: TPW = Total Plant Weight; SW = Shoot Weight; RW = Root Weight; SH = Shoot Height; RL = Root Length; Colon % = Mycorrhizal Colonization Percentage; AM = Arbuscular Mycorrhizae; EcM = Ectomycorrhizae.

	**AM TPW**	**AM SW**	**AM RW**	**AM SH**	**AM RL**	**AM Colon %**	**EcM TPW**	**EcM SW**	**EcM RW**	**EcM SH**	**EcM RL**	**EcM Colon %**
**(Intercept)**	0.00	0.00	0.00	0.00	0.00	0.00	0.00	0.00	0.00	0.00	0.00	0.00
	(0.07)	(0.08)	(0.10)	(0.07)	(0.14)	(0.10)	(0.10)	(0.14)	(0.11)	(0.18)	(0.10)	(0.18)
**Bacteria Only**	0.71 ***	0.61 ***	0.65 ***	0.53	0.74 ***	0.44 **	0.46 **	0.64 **	0.41 *	0.56 *	0.56 **	0.41
	(0.10)	(0.11)	(0.15)	(0.11)	(0.19)	(0.15)	(0.14)	(0.20)	(0.16)	(0.26)	(0.15)	(0.26)
**Fungi Only**	0.77 ***	0.85 ***	0.88 ***	0.55 ***	0.56 *	3.34 ***	0.54 ***	0.84 ***	0.58 ***	0.34	0.22	3.15 ***
	(0.11)	(0.12)	(0.16)	(0.13)	(0.22)	(0.15)	(0.14)	(0.21)	(0.17)	(0.26)	(0.15)	(0.26)
**Bacteria + Fungi**	-0.19	-0.17	-0.35	-0.22	-0.41	-0.13	-0.07	-0.17	0.12	3.60 ***	0.19	0.41
	(0.15)	(0.16)	(0.22)	(0.17)	(0.30)	(0.22)	(0.20)	(0.30)	(0.23)	(0.38)	(0.21)	(0.37)
**N**	305	250	230	171	87	280	145	101	102	55	21	89
**R2**	0.35	0.36	0.23	0.29	0.22	0.77	0.25	0.31	0.33	0.86	0.74	0.80

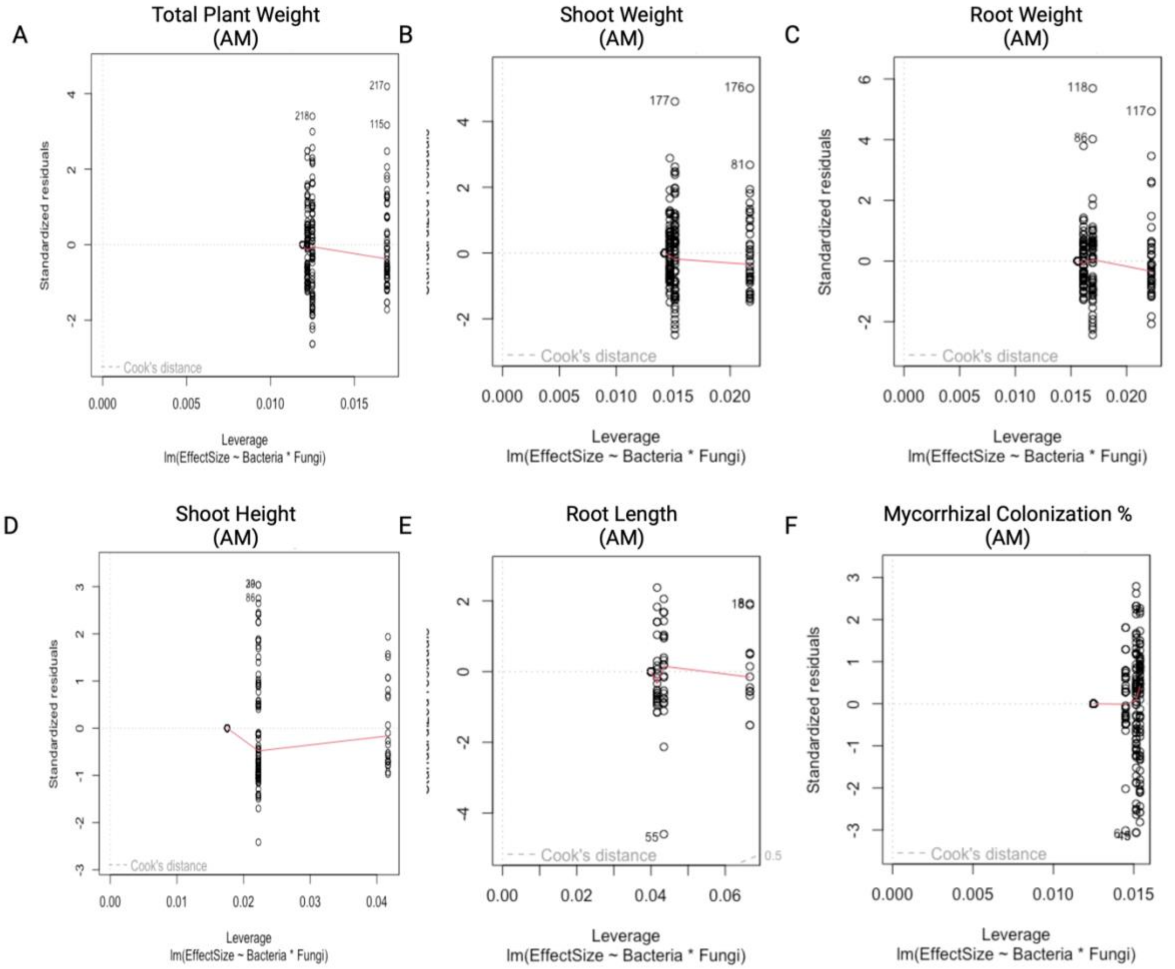

Fig. S1. Standardized residual by leverage plots for linear regression models that predict plant response effect sizes based on the presence of bacteria, arbuscular mycorrhizal (AM) fungi, and the interaction between the two. Points that fall outside of Cook's distance suggest influential or biased data points.

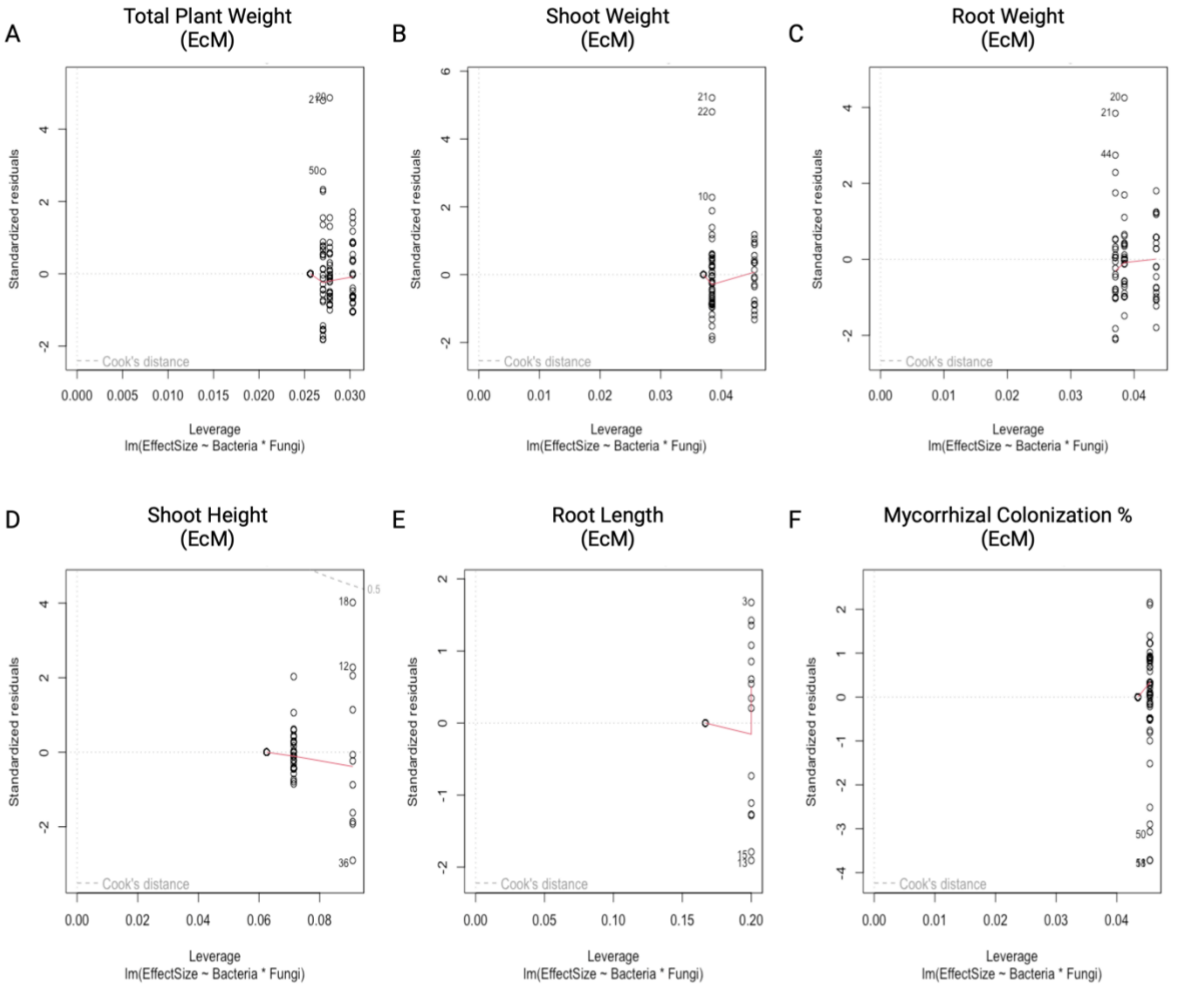

Fig. S2. Standardized residual by leverage plots for linear regression models that predict plant response effect sizes based on the presence of bacteria, ectomycorrhizal (EcM) fungi, and the interaction between the two. Points that fall outside of Cook's distance suggest influential or biased data points.
